# Dispersal of female and male *Aedes aegypti* from discarded container habitats using a stable isotope mark-capture study design in South Texas

**DOI:** 10.1038/s41598-020-63670-9

**Published:** 2020-04-22

**Authors:** Jose G. Juarez, Selene Garcia-Luna, Luis Fernando Chaves, Ester Carbajal, Edwin Valdez, Courtney Avila, Wendy Tang, Estelle Martin, Roberto Barrera, Ryan R. Hemme, John-Paul Mutebi, Nga Vuong, E. Brendan Roark, Christopher R. Maupin, Ismael E. Badillo-Vargas, Gabriel L. Hamer

**Affiliations:** 1 Department of Entomology, Texas A&M University, College Station, Texas United States of America; 2 Instituto Costarricense de Investigación y Enseñanza en Nutrición y Salud (INCIENSA), Tres Ríos Cartago, Costa Rica; 3 Entomology and Ecology Activity, Dengue Branch, Centers for Disease Control and Prevention, San Juan, Puerto Rico United States of America; 4 Centers for Disease Control and Prevention, Fort Collins, Colorado, United States of America; 5 Stable Isotope Geosciences Facility, Department of Geography, Texas A&M University, College Station, Texas United States of America; 6 Department of Entomology, Texas A&M AgriLife Research, Weslaco, Texas United States of America

**Keywords:** Ecological epidemiology, Ecological modelling

## Abstract

*Aedes aegypti* is the main vector of arboviral diseases such as dengue, chikungunya and Zika. A key feature for disease transmission modeling and vector control planning is adult mosquito dispersal. We studied *Ae aegypti* adult dispersal by conducting a mark-capture study of naturally occurring *Ae. aegypti* from discarded containers found along a canal that divided two residential communities in Donna, Texas, USA. Stable isotopes were used to enrich containers with either ^13^C or ^15^N. Adult mosquitoes were collected outdoors in the yards of households throughout the communities with BG Sentinel 2 traps during a 12-week period. Marked mosquito pools with stable isotopes were used to estimate the mean distance travelled using three different approaches (Net, Strip or Circular) and the probability of detecting an isotopically marked adult at different distances from the larval habitat of origin. We consistently observed, using the three approaches that male (Net: 220 m, Strip: 255 m, Circular: 250 m) *Ae. aegypti* dispersed further in comparison to gravid (Net: 135 m, Strip: 176 m, Circular: 189 m) and unfed females (Net: 192 m, Strip: 213 m, Circular: 198 m). We also observed that marked male capture probability slightly increased with distance, while, for both unfed and gravid females, such probability decreased with distance. Using a unique study design documenting adult dispersal from natural larval habitat, our results suggest that *Ae. aegypti* adults disperse longer distances than previously reported. These results may help guide local vector control authorities in their fight against *Ae. aegypti* and the diseases it transmits, suggesting coverage of 200 m for the use of insecticides and innovative vector control tools.

## Introduction

The mosquito *Aedes aegypti* is the primary vector of dengue, chikungunya and Zika. Diseases caused by these arboviruses place more than half a billion people at risk of infection per year globally^1^. *Aedes aegypti* is a highly anthropophilic mosquito that feeds during the day^2^ and has a tendency to exploit human man-made containers as larval habitats. With the added propensity to live in domestic and peridomestic environments *Ae. aegypti* has become a major concern for urban arboviral disease transmission^3^. A rigorous understanding of the vector ecology is critical for developing effective intervention strategies. One such feature of *Ae. aegypti* biology that has received considerable attention is adult dispersal, since it can be used to predict disease transmission and define release efforts of sterile, transgenic, or *Wolbachia* infected individuals for population suppression or replacement purposes^4,5^. Studies of mosquito dispersal are traditionally done by implementing mark-release-recapture (MRR) designs, using either laboratory reared or field captured mosquitoes^6,7^. MRR studies are done by marking external or internal body parts of mosquitoes with either dyes, fluorescent dusts, radioactive isotopes or trace elements^8^. Fluorescent dusts are the most widely used form of marking due to its cost and simplicity, since marking occurs in external body parts that can be easily checked under a UV light or epifluorescence microscope^9^. However, its simplicity comes with several costs including: low marker retention, horizontal dust transfer to unmarked individuals, and potential changes on insect behavior or survivorship^8^. Thus, an ideal marker would have lifelong retention with no influence on the biology of the mosquito.

Stable isotopic marking of mosquitoes and other insects has emerged in recent years, offering several unique advantages to help understand adult mosquito dispersal^10^. Stable isotopes are non-radioactive, non-toxic and occur naturally in the environment. These elements have atoms with the same number of protons but different number of neutrons from the more common form of the element found in nature resulting in a different mass, thus, they can be easily distinguished. For example, a stable isotope of nitrogen (^15^N) and carbon (^13^C) account for 0.732% and 1.08% of all nitrogen and carbon, respectively^11^. By enriching larval habitats with either ^15^N or ^13^C, we are elevating this rare form of element well above natural abundance levels which can serve as a label to differentiate the enriched mosquitoes from those that develop in unenriched environments. Stable isotopes have been widely used for identification purposes of different insects such as moths^12^, tsetse flies^13^, fruit flies^14^ and mosquitoes^15–17^, and studies have shown minimal impacts on their physiology, behavior and ecology^18,19^. Stable isotopic marking of *Anopheles gambiae* s. l., *Culex quinquefasciatus* and *Aedes* spp. mosquito larvae has been done before^16,17^, and used for mark-capture studies of adult mosquito dispersal^15,20^. MRR studies have been used to understand how laboratory reared mosquitoes behave under field conditions^21,22^, as well as for F1 progeny collected from field populations^23^. Most studies of *Ae. aegypti* dispersal have focused on females due to their importance for pathogen transmission^24^. However, new methods of vector control focusing on the release of males has sparked a need for an improved understanding of male dispersal ecology^6,15,16,25^.

The objective of this study was to utilize a stable isotope mark-capture design to identify dispersal of adult female and male *Ae. aegypti* in South Texas. We conducted isotopic enrichment of discarded containers (tires, small/medium/large containers and >151 L containers) along a canal in a community in Donna, Hidalgo County, Texas employing a novel study design. The marking of mosquitoes with stable isotopes was done in larval habitats, thus providing insight into dispersal of naturally occurring mosquitoes from existing larval habitats, avoiding any spurious estimation of dispersal metrics related to releasing laboratory reared adult mosquitoes. We present the mean distance travelled (MDT) and the probability of detecting marked individuals at different distances from the larval habitat of origin for male, unfed female, and gravid female *Ae. aegypti*. Our results show that naturally occurring male *Ae. aegypti* disperse further than gravid and unfed females. We suggest that this stable isotope mark-capture study design is appropriate for application to *Ae. aegypti* elsewhere in the world, depending on cost and availability of the equipment for the isotopic analysis^10^. The information derived from this type of studies is valuable to guide local vector control activities, as illustrated by inferences specific to our study site.

## Results

### Discarded containers for isotopic enrichment

During the eight-week period of mosquito sampling and isotopic marking we detected a total of 94 containers, of which 68 were enriched with ^13^C and 26 with ^15^N. We detected 82 containers (^13^C = 58; ^15^N = 24) from week 37 to 40. On week 41 we found 10 new containers (^13^C = 8; ^15^N = 2), and on week 43 we found 2 new containers (^13^C = 2). During the first week of surveillance we had four containers that were intentionally removed by a neighbor. The remaining containers persisted throughout the entire enrichment period (n = 90). However, these containers were not homogenous regarding the volume of accumulated water (or if the container became dry) and larvae/pupae presence (Table 1). We observed that over 70% of all pupae were found in tires and medium size containers (see Supplementary Fig. S1). The lowest percentage of larvae/pupae detected in containers was on week 37 (larvae = 15%; pupae = 3%), and these percentages peaked on week 40 (larvae = 67%; pupae = 47%). The increase in mosquito presence had a strong correlation (r_s_ = 0.83, p = 0.01) with precipitation in Hidalgo County. We were unable to conduct the isotopic marking of containers in week 42, due to heavy rains (138cm^3^) that occurred the previous week that flooded large sections of the communities preventing access to the study site.

**Table 1:** The number of enriched containers found in the canal of La Piñata, Donna, split by week of surveillance. No. of containers with water, (no. of containers with larvae) and [no. of containers with pupae].

Week	Tire	Tractor tire	Small container (<3 L)	Medium container (4–50 L)	Large container (51–150 L)	>151 L
37	20 (3) [1]	4 (1) [0]	3 (0) [0]	7 (1) [0]	0 (0) [0]	2 (0) [0]
38	17 (7) [2]	1 (1) [1]	5 (2) [0]	7 (5) [4]	2 (1) [1]	0 (0) [0]
39	38 (14) [5]	6 (3) [1]	5 (3) [2]	10 (7) [7]	1 (1) [0]	1 (1) [0]
40	39 (25) [16]	5 (4) [2]	7 (5) [3]	10 (8) [8]	1 (1) [1]	2 (0) [0]
41	36 (21) [9]	5 (5) [3]	5 (4) [2]	11 (8) [6]	1 (1) [1]	1 (0) [0]
43	20 (8) [5]	3 (3) [2]	2 (0) [0]	8 (4) [1]	2 (1) [1]	0 (0) [0]
44	14 (5) [1]	1 (1) [1]	1 (1) [0]	5 (2) [2]	1 (1) [0]	0 (0) [0]
45	11 (6) [3]	1 (1) [0]	1 (0) [0]	4 (2) [2]	1 (1) [1]	0 (0) [0]

### *Ae. aegypti *adult sampling and isotopic enrichment

We captured a total of 4,763 *Ae. aegypti* mosquitoes of which 2,007 were males and 2,756 females (Unfed: 1,948; Gravid: 664 and Bloodfed:144). These mosquitoes were pooled into 1,199 samples (LP: 920 and TB: 279) (Table 2), with a total of 114 isotopically marked pools detected. We detected 83 marked pools in LP (^15^N = 45 and ^13^C = 38) and 31 marked pools in TB (^15^N = 19 and ^13^C = 12). Isotopically marked (^15^N and ^13^C) individuals were found in almost all areas of LP and TB, with the exception of the last 50 m sector of LP (400–450 m) and the last two 50 m sectors of TB (300–400 m). The minimum and maximum enrichment rate (Min and Max ER) for LP was 2.12–9% and for TB 2.72–11%. Each sampling event was used to estimate the MDT of *Ae. aegypti* (male, unfed female and gravid female) using three different methods named the Net, Strip and Circular approaches.

**Table 2:** Total counts of male and female (unfed and gravid) *Ae. aegypti* mosquitoes collected, pooled and tested for isotopic enrichment in the communities of La Piñata and Tierra Bella, Donna, Texas (Percentage of total counts).

Community	Condition	No. of mosquitos	No. pools	^15^N positive	^13^C positive	Max ER	Min ER
La Piñata	Male	1460 (41.8)	352 (38.3)	20 (44.4)	22 (57.9)	12	2.62
	Unfed (F)	1470 (41.6)	369 (40.1)	17 (37.8)	11 (28.9)	7.6	1.82
	Gravid (F)	583 (16.6)	199 (21.6)	8 (17.8)	5 (13.2)	11	1.92
	Total	3513 (100)	920 (100)	45 (100)	38 (100)	9	2.12
Tierra Bella	Male	547 (49.5)	122 (43.7)	7 (36.8)	7 (58.3)	12	2.05
	Unfed (F)	478 (43.2)	121 (43.4)	9 (47.4)	4 (33.3)	11	2.37
	Gravid (F)	81 (7.3)	36 (12.9)	3 (15.8)	1 (8.4)	11	3.73
	Total	1106 (100)	279 (100)	19 (100)	12 (100)	11	2.72

### *Aedes aegypti *male Mean Distance Traveled (MDT)

We observed that males had an MDT ranging from 165 m (^15^N, LP and Net approach) to 294 m (^15^N + ^13^C, TB and Strip approach), with an overall average of 241.82 m (SE = 35.54) and a maximum distance travelled of 428.45 m. When comparing the three approaches (Net, Strip and Circular) used to estimate the MDT by community we only observed a difference for sampled males in LP with a higher MDT for the Circular (95% CI = 218.7–274.0) than for the Net (95% CI = 111.9–217.5) and Strip (95% CI = 185.1–211.8) approaches (Table 3). No difference on the male MDT’s was observed in TB by isotope or approach. We also observed that ^15^N marked males in TB had a higher MDT (95% CI, Net: 220.6–352.0; and Strip: 228.0–351.7) than those sampled in LP (95% CI, Net: 111.9–217.5; and Strip: 185.1–211.8).

**Table 3:** Estimation of the mean distance traveled (MDT) of male *Ae. aegypti* using the Net, Strip and Circular approaches (95% Confidence Interval).

Community	Approach	MDT: ^15^N	MDT: ^13^C	MDT: ^15^N + ^13^C
LP	Net	164.69 (111.9–217.5)	243.71 (198.9–288.5)	196.61 (158.8–234.5)
	Strip	198.43 (185.1–211.8)	236.08 (224.1–248.1)	222.43 (211.6–233.2)
TB	Net	286.33 (220.6–352.0)	244.48 (169.5–319.4)	249.09 (196.1–302.0)
	Strip	289.50 (228.0–351.7)	250.98 (214.1–287.9)	294.26 (242.5–346.0)
LP + TB	Circular	246.34 (218.7–274.0)	254.74 (241.6–267.9)	249.57 (236.8–262.3)

### *Aedes aegypti* unfed female Mean Distance Traveled (MDT)

We observed that unfed females had an MDT ranging from 105 m (^15^N, LP and Net approach) to 263 m (^15^N + ^13^C, TB and Strip approach), with an overall average of 195.13 m (SE = 45.38) and a maximum distance travelled of 336.85 m. We detected that ^15^N unfed females in LP had a higher MDT for the Circular (95% CI = 181.7–208.9) than for the Net (95% CI = 70.9–138.1) and Strip (95% CI = 129.4–179.3) approaches (Table 4). This same pattern was also observed for ^15^N + ^13^C individuals in LP (95% CI, Circular = 189.2–206.2, Net = 105.2–164.7, Strip = 141.9–188.6). When comparing the MDT between communities we observed that ^15^N and ^15^N + ^13^C unfed females had a higher MDT in TB than in LP.

**Table 4:** Estimation of the mean distance traveled (MDT) of unfed female *Ae. aegypti* using the Net, Strip and Circular approaches (95% Confidence Interval).

Community	Approach	MDT: ^15^N	MDT: ^13^C	MDT: ^15^N + ^13^C
LP	Net	104.49 (70.9–138.1)	179.16 (137.3–220.9)	134.95 (105.2–164.7)
	Strip	154.32 (129.4–179.3)	175.72 (153.1–198.4)	165.25 (141.9–188.6)
TB	Net	238.31 (176.5–300.1)	223.41 (82.4–364.4)	248.72 (197.2–300.3)
	Strip	238.43 (204.1–272.8)	232.05 (198.2–265.9)	263.39 (226.6–300.1)
LP + TB	Circular	195.28 (181.7–208.9)	175.10 (159.2–191.0)	197.69 (189.2–206.2)

### *Aedes aegypti* gravid female Mean Distance Traveled (MDT)

We observed that gravid females had an MDT ranging from 121 m (^15^N + ^13^C, LP and Net approach) to 217 m (^13^C, LP + TB and Circular approach), with an overall average of 155.2 m (SE = 27.06) and a maximum distance travelled of 254.9 m. We did not detect a difference on the MDT’s estimation obtained by community, isotope or approach used (Table 5).

**Table 5:** Estimation of the mean distance traveled (MDT) of gravid female *Ae. aegypti* using the Net, Strip and Circular approaches (95% Confidence Interval).

Community	Approach	MDT: ^15^N	MDT: ^13^C	MDT: ^15^N + ^13^C
LP	Net	123.78 (46.3–201–3)	131.07 (38.1–224.1)	121.36 (68.9–173.8)
	Strip	154.32 (129.4–179.3)	175.72 (153.1–198.4)	165.25 (141.9–188.6)
TB	Net	148.55 (98.9–198.2)	129.36 (-)	148.56 (98.9–198.2)
	Strip	150.99 (121.7–180.3)	143.5 (-)	187.49 (147.5–227.5)
LP + TB	Circular	142.56 (119.9–165.2)	216.46 (195.4–237.5)	189.05 (174.8–203.3)

(-) Only one gravid female was collected, no CI were calculated.

### Probability of detecting isotopically marked *Ae. aegypti*

**Figure 1:**
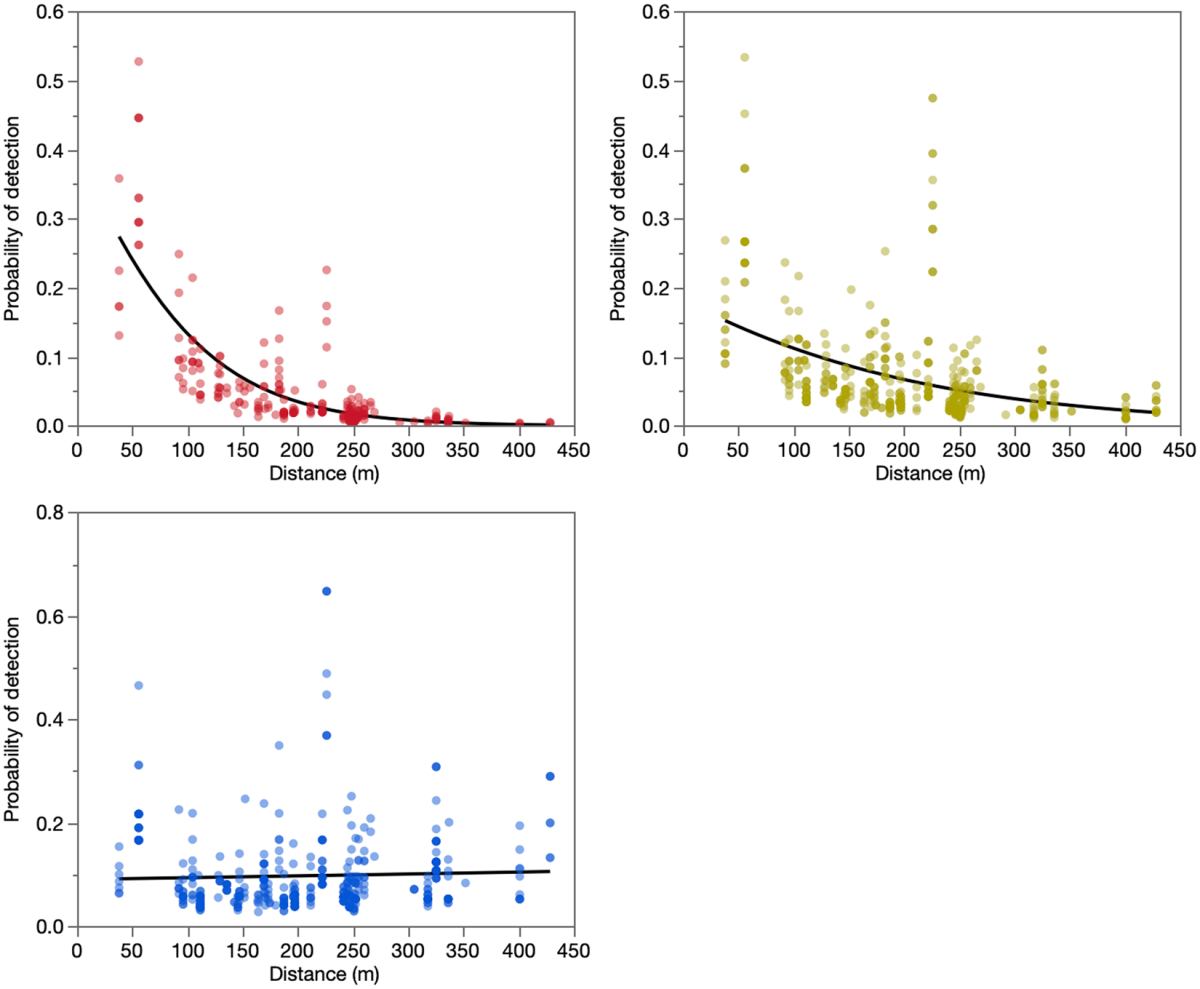
Isotopically marked *Ae. aegypti* Gravid Females (Red), Unfed Females (Yellow) and Male (Blue) pool detection probability estimated via a generalized linear mixed effects binomial model for mosquitoes captured in La Piñata and Tierra Bella, Hidalgo County, Texas, USA.

A formal goodness of fit test indicated the model was appropriate (Pearson Chi^2^/df = 0.89), furtherly, while the variance explained by the fixed factors, estimated with marginal R^2 ^amounted to one quarter of the variance (R^2^_GLMM(m)_= 0.2616), it increased to 38% of the variance when also considering the impact of the random factors through the conditional R^2^ (R^2^_GLMM(c)_ = 0.3845)^26^. The best fit model for our data had an AIC of 632.3, with an interaction between mosquito condition and distance, and sampling week as a continuous covariate, all of which were fixed effects, with a random effect for trap location (Table 6). This model considering sampling week as a fixed factor out performed a similar model where sampling week was a random factor. We estimated a positive slope close to zero (0.005779, SE = 0.002) for the probability of detection males by distance. For unfed and gravid females, negative slopes were estimated (see Supplementary Table S1). The probability curves showed how males and females dispersed through the communities. We were able to confirm that males were more likely to be detected as distance increased, by contrast to what we observed with females (Fig. 1). The results also showed that gravid females had a higher probability of detection at smaller distances than unfed females.

**Table 6:** Binomial generalized linear mixed model parameter selection for the probability of detecting an isotopically marked *Ae. aegypti *in Donna, South Texas.

Model	Parameters in model	Random effects	AIC
1	community*condition*distance + community*condition + condition*distance + community*distance + community + condition + distance	Trap, week	641.7
2	community*condition + condition*distance + community*distance + community + condition + distance	Trap, week	643.6
3	condition*distance + condition + distance	Community(Trap), week	645.6
4	condition*distance + condition + distance	Trap, week	643.6
5	condition*distance + condition + distance + week	Trap	632.3
6	condition*distance + condition + distance	Trap	644.2

## Discussion

We conducted a stable isotope mark-capture study to identify the dispersal of naturally occurring male and female (unfed and gravid) *Ae. aegypti *in South Texas. We consistently detected that male *Ae. aegypti* had a higher dispersal than gravid and unfed females, a robust result according to all approaches we used for dispersal estimation. We detected a MDT of 242 m for males and 195 m for females, higher than for some previously reported studies^6,7,23,27^. However, the maximum distance travelled of 429 m for males and 337 m for females fell short when compared with results observed in other published studies^28–31^. Our detection probability for males suggests that males continued to disperse beyond the distance of the farthest trap which we were unable to detect.

In contrast to traditional MRR studies, we relied on the natural recruitment of adult mosquitoes from discarded container larval habitats found on the west bank of a canal that bordered two communities in South Texas. We observed that 84% of all the containers found throughout the eight weeks of enrichment were found in the first week. It appears that this area was not used as a consistent dumping site by community members, since garbage collections were seen within the community on a weekly basis. The west bank of the canal accumulated discarded containers and used tires accounted for more than half of all containers found. This might be because disposal of tires in Donna, Texas, needs to be done in an authorized landfill. Generally these landfills only accept two tires/household/month for free, with an additional a $5–8 cost per additional tire, an expensive price considering the income for residents of this area^32^. Tires and medium size containers played a key role during the enrichment procedures since, on average, 5.25 tires/week and 3.75 medium size containers/week had pupae. We observed that mosquitoes produced by the discarded containers allowed us to detect a minimum–maximum ER of 2.05–12% for male and 1.82–11% for female pools of adult *Ae. aegypti*. If we use the ER as a proxy for recapture, we have similar results with other MRR studies that used fluorescent dusting or paints as a marking method^6,7,21,23,31^. However, recapture methods between studies varied making comparisons difficult. Further studies are needed to evaluate the survival and fitness of isotopically enriched mosquitos as a marking tool for mosquito control methods relaying on male releases.

The dispersal analysis of our study showed that *Ae. aegypti* female (unfed and gravid) MDT was 60 m (average: 146 m in LP and 212 m in TB) less than what we observed for males (average: 210 m in LP and 272 m in TB). MRR studies based on laboratory reared male and female *Ae. aegypti *have generally showed limited dispersal distances with MDT’s averaging 50–100 m^6,7,23,27^. Even with this stable isotope mark-capture study design, we expected males to have similar MDT’s to those found in females, something that we did not observe. Interestingly, more recent studies have shown the ecological plasticity of male *Ae. aegypti *with MDT’s ranging from 44 to 575 m depending on the time of year of collection^31^. This highlights the importance of seasonal environmental conditions for the movement of this mosquito species, and an aspect of *Ae. aegypti* dispersal deserving further study. When analysing the probability of detecting a marked pool at different distances from the larval habitat, we observed that males had an increased probability of detection at a higher distance when compared to females. These results suggest that if traps were set at further distances, we might still be able to certainly detect male dispersion. Similar to other studies on female dispersion, we observed that the probability of detection has a steep decrease after 100 m. These results show that naturally occurring male *Ae. aegypti *will disperse farther than unfed and gravid females. This observation of males dispersing farther than females is consistent with the prior stable isotope mark-capture study of *Ae. albopictus* in Texas^15^.

When comparing the communities, we observed using the Net and Strip approaches that unfed female *Ae. aegypti *had a higher dispersion in TB than in LP. Even with no statistical difference for the probability of detecting an isotopically marked adult between communities, we believe that the uniformity of the community needs to be taken into consideration for dispersal studies. It has been observed that size and density of oviposition habitats influence the dispersal of *Ae. aegypti*^33,34^. This might have been our scenario since the east bank of the canal (the side on TB) had fewer discarded containers and households in this community were double in size than those found in LP.

In regard to our study design, we acknowledge that the enrichment of discarded containers to mark naturally occurring mosquitos makes comparisons with other MRR studies complicated and need to be interpreted carefully. Some limitations of our study design are that we cannot cross-reference a specific container with a specific marked pool and the exact day of emergence is unknown for captured marked adults. In addition, the container transect receiving isotope enrichment was at the edge of the community along a canal which was 20 m away from BG Sentinel 2 traps. We also did not include climatic variables into our model due to the uncertainty of when the captured marked mosquitos emerged from the enriched larval habitats. Nonetheless, we consider our results to be a good estimation of how naturally occurring *Ae. aegypti* disperse within these communities in South Texas.

We were able to show a successful isotopic marking and detection of naturally occurring adult *Ae. aegypti*. This approach to studying mosquito movement which capitalizes on isotopic marking of naturally existing larvae in diverse container habitats provides advantages over alternative methods for conducting MRR studies. We believe this methodology can be applied to *Ae. aegypti* elsewhere in the world, if costs for isotope analysis are taken into consideration ($6 per sample in our case). This methodology may be used to address multiple questions related to the biology and control of mosquitoes in local settings. Our results show that vector control programs that target *Ae. aegypti* in the Lower Rio Grande Valley should consider the operational implications of *Ae. aegypti* having the ability to emerge in one community and disperse to an adjacent community. In addition, the application of adulticides or other innovative intervention tools targeting *Ae. aegypti* around the home of locally acquired human cases would benefit from coverage out to 200 m.

## Materials and methods

### Study site

**Figure 2:**
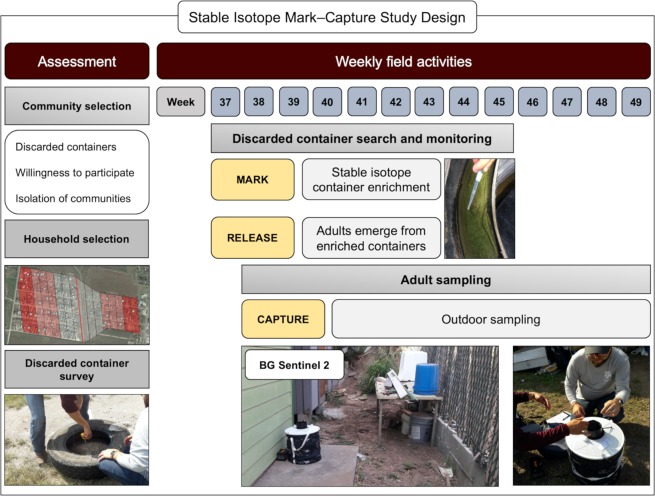
A mark–capture study design for the isotopically enrichment of naturally occurring *Ae. aegypti*. We carried out an initial assessment of communities located in Hidalgo County, based on the presence of discarded containers in public property, willingness of community members to participate and isolation of the communities. Houses were selected based on distance to the discarded container larval habitats of enrichment. Isotopic enrichment of larval habitats was carried out on a weekly basis from the 37 to the 45^th^ week of 2017. Weekly adult sampling was done using BG Sentinel 2 traps, set outside of the house from week 38 to 49^th^ of 2017. The map was developed using QGIS 3.4.4 (https://qgis.org/en/site/) with Map data: Google, Maxar Technologies.

We evaluated different communities in the region to determine the most appropriate location for an isotopic mark-capture study design (Fig. 2). We based our study site selection on the ability to have access to freely discarded containers (those found in public property), willingness of community members to participate in the sampling efforts, and isolation from other communities. The study took place from September 5^th^ to December 7^th^, 2017, in the communities of La Piñata (LP) and Tierra Bella (TB) (26°7′43.78″ -98°3′19.63″) in Donna, Hidalgo County, Texas (Fig. 2). The study area consisted of a total of 23.4 ha (LP = 15 and TB = 8.4) with 180 houses (LP = 136 and TB = 44). Housing density (mean ± S.E.: 7.15 ± 2.7) by community was LP = 9.1 houses/ha and TB = 5.2 houses/ha. Each occupied house in the study area was georeferenced using an eTrex20x GPS (Garmin, USA). In 2017, the city of Donna had a population of 16,638, 92% of whom were Hispanic or Latino. Thirty five percent of the population lives below the poverty line, 30% of the people under 65 years have no health insurance and 19% are foreign born individuals^35^. Geographically, the communities of LP and TB are surrounded by agricultural fields, which were not cultivated during the study period. The two communities are divided by a 25-meter-wide canal. The temperature in the region is considered cold/dry from November to February (7–21 °C), and hot/dry from March to October (22–40 °C), with a rainy season starting in April, peaking in September and finishing in October (average annual: precipitation 63.5 cm and relative humidity 75%)^36^.

### Discarded container search and monitoring

**Figure 3:**
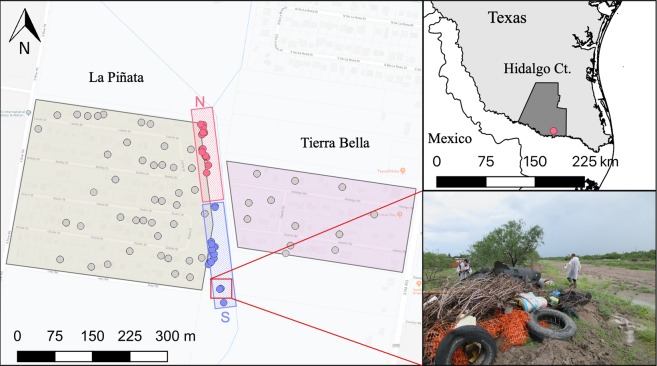
Location of the communities of La Piñata and Tierra Bella, Donna, in the County of Hidalgo, Texas. The boundary of La Piñata is enclosed by the beige area to the left and the boundary of Tierra Bella is enclosed in the pink area to the right. Gray dots = houses with weekly surveillance of mosquitoes, blue dots = larval habitats with ^13^C isotope enrichment, and red dots = larval habitats with ^15^N isotope enrichment. The red square with the above N= north transect and the blue square with lower S= south transect. The map was developed using QGIS 3.4.4 (https://qgis.org/en/site/), Map data: Google Maps, and with publicly available administrative boundaries (https://gadm.org/license.html).

We performed a preliminary assessment of discarded containers on the west and east banks of the canal that separated the communities (Fig. 3), no containers were found on the east bank of the canal. We estimated the average ±SD no. of pupae/container was 1.65 ± 0.6 a number similar to that observed in communities in Mexico^37^. Monitoring and marking of containers were done on a weekly basis from September 11^th^ to November 8^th^, by the same team members. Container counting and marking started one week before (week 37) adult mosquito sampling (week 38). We sampled a transect of 400 m of public property next to a canal that divides the communities of LP and TB and searched for all containers capable of holding water (Fig. 3). Each container was uniquely labelled with an oil-based marker. Records were kept for the type of container, amount of water found, presence of larvae/pupae, amount of isotope added and GPS coordinates. The labelling of containers allowed us to track if containers were removed, needed enrichment or if new ones arose. We had four isotopically marked containers that were removed during the first week of isotopic marking. These containers were not taken into consideration for the dispersal analysis given insufficient time to generate marked adults.

### Household selection for adult sampling

**Figure 4:**
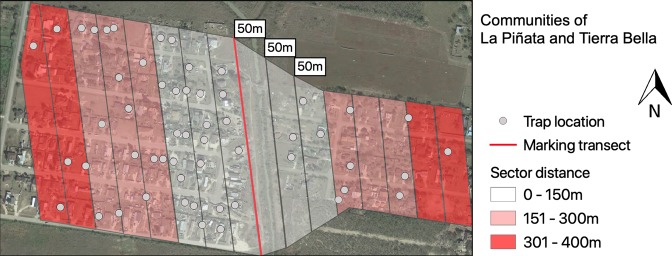
Communities of La Piñata and Tierra Bella divided into sectors for BG2 surveillance. Sectors were divided based on the distance to the isotopically marked larval habitat transect. The map was developed using QGIS 3.4.4 (https://qgis.org/en/site/) with Map data: Google, Maxar Technologies.

The communities were divided into three sectors (1^st^: 0–150 m; 2^nd^: 151–300 m; and 3^rd^: 301–400 m) based on parallel proximity to the discarded container transect enriched with stable isotopes for mosquito marking (Fig. 4). The number of houses for weekly sampling was based on housing density and distance to the isotopically marked larval habitat transect. We randomly selected 28 houses from LP (1.8 house/ha) and 12 from TB (1.5 house/ha), to have a similar sampling effort in each community^6^. We deployed 50% of all traps in the 1^st^ sector (0–150 m), 30% in the 2^nd^ (151–300 m) and 20% in the 3^rd^ (301–400 m) for LP. The distribution of traps in TB was 30% in the 1^st^ sector, 50% in the 2^nd^, and 20% in the 3^rd^; the selection constrained by household participation. Our trap distribution was designed to maximize our capture success based on previous MRR studies of *Ae. aegypti* (0.35% to 8% recaptures) were over 80% of recaptures happened in the first 100 m^6,21,23,24^. In the statistical analysis section, we further explain how we took into consideration trap density for our different models.

### Stable isotope enrichment and adult marking

The isotopically marked larval habitat transect was divided into two sections of 200 m each (Fig. 2). All of the containers with water in the south transect were enriched with D-Glucose (U-13C6, 99%) (^13^C) (Cambridge Isotope Laboratories, USA) and in the north transect with Potassium Nitrate (^15^N, 99%) (^15^N) (Cambridge Isotope Laboratories, USA). This step corresponds to the marking and releasing in an MRR study but the current study design marks larval mosquitoes naturally occurring in the field which remain marked as adults. Isotopic marking was done using a concentration of 0.002 g/L for both isotopes which was based on previous studies marking larval habitats of *Culex* mosquitoes^15,20^ as well as, optimal isotopic marking concentrations based on laboratory-reared *Ae. aegypti*^38^. During the first isotopic marking, each container received a full dose of isotopes. Subsequently only half doses were added to each container, unless a rain event occurred that added water to the containers in which case a full isotope dose was used again. For quality assurance and to guide our enrichment procedures, on a weekly basis we randomly selected one container that had pupae from both transects, collected three individuals and allowed them to emerge as adults in the laboratory in Weslaco, TX. These mosquitoes were then transported in coolers with dry ice to our laboratory in College Station, TX, to be processed for the stable isotope analysis.

### Adult sampling

We carried out weekly outdoor collections in LP (week 38) and TB (week 39) using BG Sentinel 2 traps baited with BG-Lure (Biogents, Germany) (artificial skin odor based on a mixture of ammonia, lactic acid and caproic acid) which was replaced every 60 days. Trap deployment was done between 9:00 and 10:00 am, traps were left for about 23 h, picked up the next day from 8:00 to 9:00 am. To prevent mosquito damage, collection bags were placed in a plastic container inside a cooler with icepacks. Mosquitoes were classified by sex (male or female), physiological state (unfed or gravid) and identified to species^39,40^ (see Supplementary Dataset S1). We separated the mosquito samples in pools with a maximum of five (male and unfed female) and four (gravid females) mosquitoes for each given species or groups^17^. Blood-fed females were excluded from the samples for this study, since they were used for bloodmeal analysis in a different study. All samples were stored at −80 °C and transported in coolers with dry ice to our main laboratory in College Station, Texas, for further analysis.

### Stable isotope analysis

Collected adults were analyzed to identify which specimens were uniquely enriched with stable isotopes. Male and female (unfed and gravid) *Ae. aegypti* samples were placed in tin capsules (Tin capsules, Costech, Valencia, CA, USA) arranged in a 96-well cell culture plate, desiccated at 56 °C for 18–24 h, and then sealed by hand into spherical balls. Plates with samples were submitted to the Stable Isotope Geosciences Facility, Texas A&M University, College Station, Texas, for dual ^15^N and ^13^C analysis using a procedure previously described^15^. Briefly, the analysis was carried out using a Carlo Erba NA 1500 Series 2 Elemental Analyzer (EA) attached to a Thermo-Finnigan Conflo III and a Thermo Finnigan Delta Plus XP isotope ratio mass spectrometer (IRMS). The process consists of combusting the samples at 1,200 °C which will pass through two reactors to convert the nitrogen oxides generated in the oxidation reactor to N_2_ gas. The CO_2_ and N_2_ gases generated are separated chromatographically and analyzed on the IRMS.

### Statistical analysis

Isotopically enriched discarded containers were tested for a correlation between precipitation and the presence of larvae/pupae in these isotopic enriched larval habitats using Spearman’s ρ. To evaluate the capture rates of isotopically marked *Ae. aegypti *pools we calculated the Maximum (Max ER; No. positive pools/Total pools tested) and Minimum Enrichment Rates (Min ER; estimated using PooledInfRate (Biggerstaff, CDC, www.cdc.gov/ncidod/dvbid/westnile/software.htm)). The Max ER assumes that all mosquitoes in an enriched pool were isotopically marked, while the Min ER assumes that only one mosquito was isotopically marked. To estimate the Mean Distance Travelled (MDT) and the probability of detection of an isotopically enriched mosquito, we measured the distance (mean, min, max and standard error) between the geographic coordinates of each enriched larval habitat and each BG Sentinel 2 trap. Distances were measured using the distance matrix function in QGIS 3.4.4 (QGIS development team 2019). For this, we assumed that isotopically marked mosquitoes had the same probability of emerging from any larval habitat with the same isotopic enrichment.

**Figure 5:**
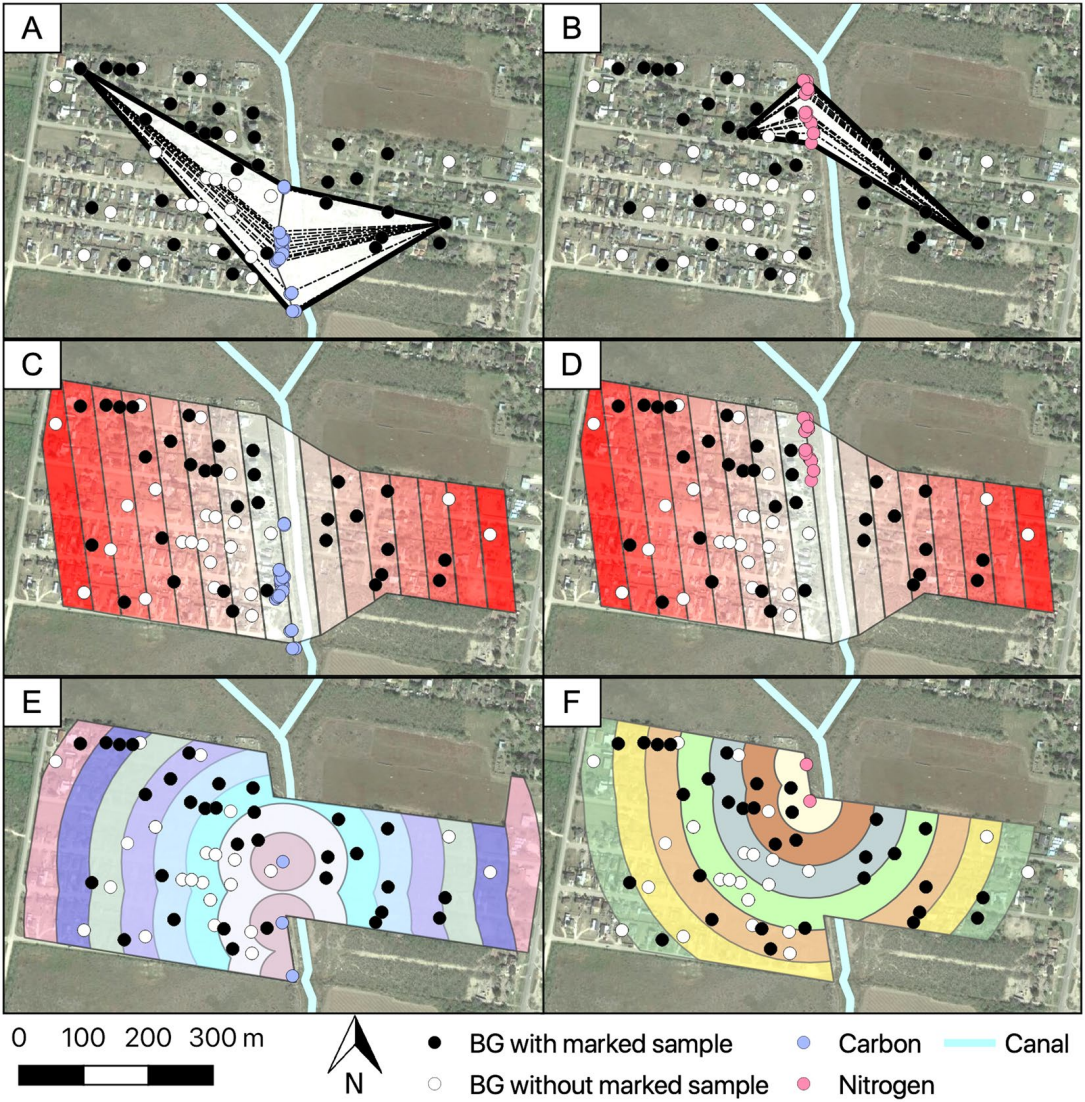
Mean distance traveled approaches used for the estimation of the natural dispersion of isotopically marked mosquitoes. (**A,B**) Net approach measurements were based on the mean distance of the house with a marked sample to every larval source enriched. (**C,D**) Strip approach and (**E,F**) Circular approach averages the max and min distance for all houses per sector, taking into account indirect flight patterns and trap densities. Dotted lines = distance from larval enriched source to house with marked sample. The map was developed using QGIS 3.4.4 (https://qgis.org/en/site/) with Map data: Google, Maxar Technologies.

We estimated the MDT using three different and independent approaches which we called Net, Strip and Circular^15,20^. The Net approach estimates MDT as the linear dispersion of a given mosquito from any possible source of isotopic marking to the trap where it was captured, without accounting for indirect flight patterns and trapping effort (Fig. 5A,B)^23^. The Strip and Circular approaches follow a procedure based on Morris^9^, where the area contiguous to the release is divided in sectors and annuli with 50 m increments. These area divisions account for indirect flight patterns and compensate for unequal trapping efforts^20,41^. For both the Strip and Circular approaches we also made the assumption that adult *Ae. aegypti* movement from the isotopically marked larval habitats was isotropic with similar movement where we sampled and where we did not (the adjacent agricultural fields)^42^. The Strip approach assumes a one-dimensional diffusion^43^ from the enriched larval habitats to the trap where the marked pool was detected, taking into consideration the area of each sector (Fig. 5C,D). The Circular approach (standard procedure) adapted the annuli method, which assumes a two-dimensional diffusion^43^. For this, we defined five clusters of enrichment (^13^C = 3 and ^15^N = 2) –for the larval habitats in the transect– using the k-means clustering method in R3.2 (Vienna, Austria)^44^. K-means method uses the nearest mean distances between larval habitats to identify high-density regions that allows the choice of an optimal number of clusters^45^. The Circular approach uses the distance between marked pools and clusters of larval habitats, taking into account the area of each annuli (Fig. 5E,F).

We estimated the probability of detecting isotopically marked *Ae. aegypti* pools using binomial generalized linear mixed models^46^. Briefly, we started by considering a full model described by the following equation:

1 $$Log(\pi /1-\pi )=\mu +{\alpha }_{i}+{\gamma }_{j}+{\beta }_{1}{x}_{k}+{\beta }_{2}({\alpha }_{i}{\gamma }_{j})+{\beta }_{3}({\alpha }_{i}{x}_{k})+{\beta }_{4}({\gamma }_{j}{x}_{k})+{\beta }_{5}({\alpha }_{i}{\gamma }_{j}{x}_{k})+{\pi }_{l}+{\tau }_{m}+{{\epsilon }}_{ijlmk}$$

Where fixed factors included: μ the intercept, a parameter $\alpha$ accounts for the community where adult mosquitoes were sampled, and had two *i* levels (LP or TB), mosquito condition (denoted by $\gamma$) had three *j* levels (male, gravid, or unfed), the mean distance from the enriched larval habitats was a covariate for each *k* observation, whose effect was measured by parameter ${\beta }_{1}$. Parameters ${\beta }_{2},{\beta }_{3},{\beta }_{4}$ accounted, respectively, for the interaction between community and condition; community and distance, condition and distance, while parameter ${\beta }_{5}$ accounted for the three-way interaction between community, condition and mean distance travelled. Meanwhile, the model considered a categorical variable with unique ids for each *l* trap ($\pi$) and a variable for the *m* weeks ($\tau$) when mosquitoes were sampled as random factors. These random factors were included to account for spatial effects associated with trap location and the repeated sampling over the study period. The random factors were assumed to follow an identical and independent normal distribution:

2 $$\pi  \sim N(O,{\sigma }_{\pi }^{2})$$

And

3 $$\tau  \sim N(O,{\sigma }_{\tau }^{2})$$

where ${\sigma }_{\pi }^{2}$ and ${\sigma }_{\tau }^{2}$ are the variance for the trap and sampling week random factors, ${\epsilon }$ was the model error.

Models were fitted using the Laplace estimation method implemented in SAS 9.4 (GLIMMIX, SAS Institute Inc., NC, USA)^47^. The model presented in (1) was then simplified through a process of backward elimination^48^, where parameters accounting for the three-way interaction between variables, then the two-way interactions and single parameters were sequentially removed. The reduced model was selected based on the Akaike information criterion (AIC), a metric for model selection that trades off goodness of fit and parameter number^48,49^. The goodness of fit of the final model was evaluated using the conditional and marginal R^2^ values^26^ and a Chi^2^ test for GLMMs goodness of fit^50^.

## Supplementary information

Supplementary Information.

Supplementary Data S1.

## Data Availability

All datasets used for the development of this manuscript have been made available and can be found in the Supplementary Data S1.

## References

[1] WHO. *Global vector control response 2017–2030. World Health Organization* (2017).

[2] Ponlawat A, Harrington LC (2015). Blood feeding patterns of *Aedes aegypti* and *Aedes albopictus* in Thailand. J. Med. Entomol..

[3] Eder, M. *et al*. Scoping review on vector-borne diseases in urban areas: Transmission dynamics, vectorial capacity and co-infection. *Infect. Dis. Poverty* **7** (2018). 10.1186/s40249-018-0475-7 PMC6120094 30173661

[4] Reiter P (2007). Oviposition, dispersal, and survival in *Aedes aegypti*: Implications for the efficacy of control strategies. Vector-Borne Zoonotic Dis..

[5] Perkins TA (2013). A systematic review of mathematical models of mosquito-borne pathogen transmission: 1970-2010. J. R. Soc. Interface.

[6] Valerio L, Facchinelli L, Ramsey JM, Scott TW (2012). Dispersal of male *Aedes aegypti* in a coastal village in Southern Mexico. Am. J. Trop. Med. Hyg..

[7] Russell RC, Webb CE, Williams CR, Ritchie SA (2005). Mark-release-recapture study to measure dispersal of the mosquito *Aedes aegypti* in Cairns, Queensland, Australia. Med. Vet. Entomol..

[8] Verhulst, N. O., Loonen, J. A. & Takken, W. Advances in methods for colour marking of mosquitoes. *Parasites and Vectors* **6** (2013). 10.1186/1756-3305-6-200 PMC3708792 23835091

[9] Morris, C. D., Larson, V. L. & Lounibos, L. L. Measuringmosquito dispersal for control programs. *J. Am. Mosq. Control Assoc*. 7, 608–615 (1991). 1686275

[10] IAEA. Manual for the use of stable isotopes in entomology. in *IAEA-SI*. 10.1007/978-3-658-08844-6_16 (2009)

[11] Beerling DJ, Lajtha K, Michener RH (2006). Stable isotopes in ecology and environmental science. J. Anim. Ecol..

[12] Hood-Nowotny R (2016). Stable isotope markers differentiate between mass-reared and wild Lepidoptera in Sterile Insect Technique programs. Florida Entomol..

[13] Hood-Nowotny R (2011). Intrinsic and synthetic stable isotope marking of Tsetse flies. J. Insect Sci..

[14] Botteon V (2018). Can stable isotope markers be used to distinguish wild and mass-reared *Anastrepha fraterculus* flies?. PLoS One.

[15] Medeiros MCI, Boothe EC, Roark EB, Hamer GL (2017). Dispersal of male and female *Culex quinquefasciatus* and *Aedes albopictus* mosquitoes using stable isotope enrichment. PLoS Negl. Trop. Dis..

[16] Opiyo, M. A. *et al*. Using stable isotopes of Carbon and Nitrogen to mark wild populations of *Anopheles* and *Aedes* mosquitoes in South-Eastern Tanzania. *PLoS One* **11** (2016). 10.1371/journal.pone.0159067 PMC4938253 27392083

[17] Hamer GL (2012). Evaluation of a stable isotope method to mark naturally-breeding larval mosquitoes for adult dispersal studies. J. Med. Entomol..

[18] Hyodo F (2015). Use of stable carbon and nitrogen isotopes in insect trophic ecology. Entomological Science.

[19] Hood-Nowotny R, Knols BGJ (2007). Stable isotope methods in biological and ecological studies of arthropods. Entomol. Exp. Appl..

[20] Hamer, G. L. *et al*. Dispersal of adult *Culex* mosquitoes in an urban West Nile virus hotspot: A mark-capture study incorporating stable isotope enrichment of natural larval habitats. *PLoS Negl. Trop. Dis*. **8** (2014). 10.1371/journal.pntd.0002768 PMC3967984 24676212

[21] Winskill P (2015). Dispersal of engineered male *Aedes aegypti* mosquitoes. PLoS Negl. Trop. Dis..

[22] Muir LE, Kay BH (1998). *Aedes aegypti* survival and dispersal estimated by mark-release-recapture in northern Australia. Am. J. Trop. Med. Hyg..

[23] Harrington LC (2005). Dispersal of the dengue vector *Aedes aegypti* within and between rural communities. Am. J. Trop. Med. Hyg.

[24] Guerra CA (2014). A global assembly of adult female mosquito mark-release-recapture data to inform the control of mosquito-borne pathogens. Parasites and Vectors.

[25] Bellini R (2010). Dispersal and survival of *Aedes albopictus* (Diptera: Culicidae) males in Italian urban areas and significance for Sterile Insect Technique application. J. Med. Entomol..

[26] Nakagawa S, Schielzeth H (2013). A general and simple method for obtaining R2 from generalized linear mixed-effects models. Methods Ecol. Evol..

[27] Verdonschot PFM, Besse-Lototskaya AA (2014). Flight distance of mosquitoes (Culicidae): A metadata analysis to support the management of barrier zones around rewetted and newly constructed wetlands. Limnologica.

[28] Wolfinsohn M, Galun E (1953). A Method for determining the Flight Range of *Aedes aegypti* (Linn.). Bulletin of the Research Council of Israel.

[29] Davis NC, Shannon RC (1930). The Flight of *Stegomyia aegypti* (L.) 1. Am. J. Trop. Med. Hyg.s.

[30] Liew C, Curtis CF (2004). Horizontal and vertical dispersal of dengue vector mosquitoes, *Aedes aegypti* and *Aedes albopictus*, in Singapore. Med. Vet. Entomol..

[31] Mains JW, Kelly PH, Dobson KL, Petrie WD, Dobson SL (2019). Localized Control of *Aedes aegypti* (Diptera: Culicidae) in Miami, FL, via Inundative Releases of *Wolbachia*-Infected Male Mosquitoes. J. Med. Entomol..

[32] TCEQ. Disposing of tires from your home or private property. Available at: https://www.tceq.texas.gov/tires/tires-disposal-qa/#home. (Accessed: 28th May 2019) (2018).

[33] Brown HE, Cox J, Comrie AC, Barrera R (2017). Habitat and density of oviposition opportunity influences *Aedes aegypti* (Diptera: Culicidae) flight distance. J. Med. Entomol..

[34] Edman JD (1998). *Aedes aegypti* (Diptera: Culicidae) Movement Influenced by Availability of Oviposition Sites. J. Med. Entomol..

[35] U.S.Census Bureau. Donna city, Texas. Available at: https://www.census.gov/quickfacts/fact/table/mercedescitytexas,donnacitytexas/PST045218. (Accessed: 29th January 2019) (2017).

[36] NOAA. National Weather Service: Climate Prediction Center. *Local ClimatologicalData, McAllen Miller Int Airport, TX*. Available at: https://w2.weather.gov/climate/xmacis.php?wfo=bro. (Accessed: 29th January 2019) (2017).

[37] Arredondo-Jiménez JI, Valdez-Delgado KM (2006). *Aedes aegypti* pupal/demographic surveys in southern Mexico: consistency and practicality. Ann. Trop. Med. Parasitol..

[38] Garcia-Luna, S. M. *et al*. Stable Isotope Marking of Laboratory-Reared *Aedes aegypti* (Diptera: Culicidae). *J. Med. Entomol*.**1** (2019). 10.1093/jme/tjz210 PMC7044722 31751467

[39] Darsie, R. & Ward, R. *Identification and geographical distribution of the mosquitoes of North America*, *North of Mexico*. *University Press of Florida*. 10.7589/0090-3558-43.4.806 (2005).

[40] Wilkerson RC (2015). Making mosquito taxonomy useful: A stable classification of tribe Aedini that balances utility with current knowledge of evolutionary relationships. PLoS One.

[41] Silver, J. B. Measuring adult dispersal. in *Mosquito Ecology: Field sampling methods ***53**, 1689–1699 (Springer Science+Business Media B.V., 2013).

[42] van Putten B, Visser MD, Muller-Landau HC, Jansen PA (2012). Distorted-distance models for directional dispersal: A general framework with application to a wind-dispersed tree. Methods Ecol. Evol..

[43] Okubo, A. & Levin, S. A. The basics of difussion. in *Diffusion and ecological problems: Modern perspectives* (eds. Antman, S., Marsden, J. E., Sirovich, L. & Wiggins, S.) 463 (Springer Science, 2000).

[44] Bock, H.-H. Clustering methods: A history of k-means algorithms. in *Selected contributions in data analysis and classification* (eds. Brito, P., Bertrand, P., Cucumel, G. & Carvalho, F. d.) 619 (Springer-Verlag Berlin Heidelberg, 2007).

[45] Bocard, D., Gillet, F. & Legendre, P. *Numerical Ecology with R*. (Springer Science+Business Media. 10.1007/978-0-387-78171-6 (2011).

[46] Bolker BM (2009). Generalized linear mixed models: A practical guide for ecology and evolution. Trends in Ecology and Evolution.

[47] SAS InstituteInc. PROC GLIMMIX: G-Side and R-Side Random Effects and Covariance Structures. *SAS/STAT(R) 9.22 User’s Guide*. Available at: https://support.sas.com/documentation/cdl/en/statug/63347/HTML/default/viewer.htm#statug_glimmix_a0000001405.htm. (Accessed: 15th May 2019) (2010).

[48] Faraway, J. *Linear Models with R. Text in statistical science series* (Chapman and Hall/CRC Texts in Statistical Science Series, 2005).

[49] Burnham KP, Anderson RP (2004). Multimodel inference: Understanding AIC and BIC in model selection. Sociol. Methods Res..

[50] Faraway, J. J. *Extending the Linear Model with R: Generalized Linear*, *Mixed Effects and Nonparametric Regression Models* (CRC Press, 2016).

