# Supplementary material for: Dispersal of female and male Aedes aegypti from discarded container habitats using a stable isotope mark-capture study design in South Texas
Source: Sci Rep. 2020 Apr 22;10:6803. doi: 10.1038/s41598-020-63670-9 (PMC7176680; doi:10.1038/s41598-020-63670-9)
Supplement: Supplementary file 1 — Supplementary Information. [file 41598_2020_63670_MOESM1_ESM.docx]

Dispersal of female and male *Aedes aegypti* from discarded container habitats using a stable isotope mark-capture study design in South Texas.

Jose G. Juarez^1*^, Selene Garcia-Luna^1^, Luis Fernando Chaves^2^, Ester Carbajal^1^, Edwin Valdez^1^, Courtney Avila^1^, Wendy Tang^1^, Estelle Martin^1^, Roberto Barrera^3^, Ryan Hemme^3^, John-Paul Mutebi^4^, Nga Vuong^4^, Brendan Roark^5^, Christopher R. Maupin^5^, Ismael E. Badillo-Vargas^1-6^, and Gabriel L. Hamer^1*^

^1^Department of Entomology, Texas A&M University, College Station, Texas, United States of America
^2^Instituto Costarricense de Investigación y Enseñanza en Nutrición y Salud (INCIENSA), Tres Ríos, Cartago, Costa Rica

^3^Entomology and Ecology Activity, Dengue Branch, Centers for Disease Control and Prevention, San Juan, Puerto Rico, United States of America

^4^Centers for Disease Control and Prevention, Fort Collins, Colorado, United States of America

^5^Stable Isotope Geosciences Facility, Department of Geography, Texas A&M University, College Station, Texas, United States of America

^6^Department of Entomology, Texas A&M AgriLife Research, Weslaco, Texas, United States of America

**Supplementary Information**


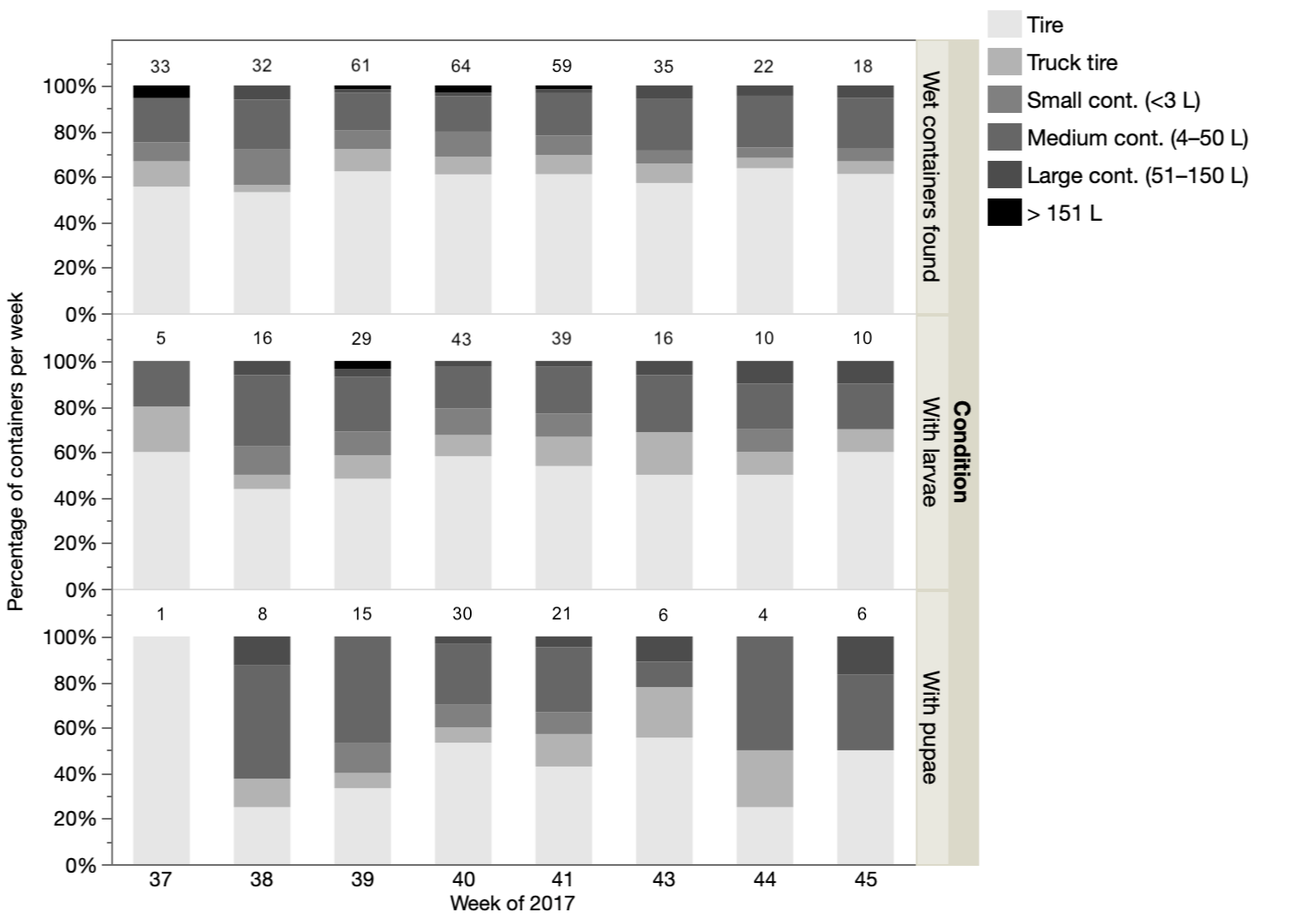


Supplemental Figure S1. Percentage distribution of the type of containers split by week of surveillance and enrichment. A) Percentage distribution of wet containers found, B) with larvae and C) with pupae. Numbers on top of bars refers to the total weekly count of containers wet, with larvae or with pupae.

Supplementary Table S1. Parameter estimates of the 5^th^ binomial generalized linear mixed model for the probability of detecting an isotopically marked *Ae. aegypti* in Donna, South Texas.

| Effect | condition | Estimate | Standard Error | DF | t Value | Pr > \|t\| |
| --- | --- | --- | --- | --- | --- | --- |
| Intercept |  | -8.8707 | 1.9150 | 54 | -4.63 | <.0001 |
| distance |  | -0.00499 | 0.002637 | 1138 | -1.89 | 0.0587 |
| condition | Gravid | 0.6657 | 0.7442 | 1138 | 0.89 | 0.3712 |
| condition | Male | -0.5964 | 0.5626 | 1138 | -1.06 | 0.2894 |
| condition | Unfed | 0 | . | . | . | . |
| week |  | 0.1637 | 0.04316 | 1138 | 3.79 | 0.0002 |
| distance*condition | Gravid | -0.00650 | 0.004499 | 1138 | -1.44 | 0.1488 |
| distance*condition | Male | 0.005779 | 0.002708 | 1138 | 2.13 | 0.0331 |
| distance*condition | Unfed | 0 | . | . | . | . |
